# Exosomes-Associated DNA—New Marker in Pregnancy Complications?

**DOI:** 10.3390/ijms20122890

**Published:** 2019-06-13

**Authors:** Barbora Konečná, Ľubomíra Tóthová, Gabriela Repiská

**Affiliations:** 1 Institute of Molecular Biomedicine, Faculty of Medicine, Comenius University in Bratislava, Bratislava 81108, Slovakia; tothova.lubomira@gmail.com; 2 Institute of Physiology, Faculty of Medicine, Comenius University in Bratislava, Bratislava 81372, Slovakia; gabika.repiska@gmail.com

**Keywords:** cell-free DNA, exosomes, extracellular vesicles, fetal DNA, preeclampsia, growth retardation, gestational diabetes mellitus

## Abstract

Despite a large number of studies, the etiology of pregnancy complications remains unknown. The involvement of cell-free DNA or fetal cell-free DNA in the pathogenesis of pregnancy complications is currently being hypothesized. Cell-free DNA occurs in different forms—free; part of neutrophil extracellular traps; or as recently discovered, carried by extracellular vesicles. Cell-free DNA is believed to activate an inflammatory pathway, which could possibly cause pregnancy complications. It could be hypothesized that DNA in its free form could be easily degraded by nucleases to prevent the inflammatory activation. However, recently, there has been a growing interest in the role of exosomes, potential protectors of cell-free DNA, in pregnancy complications. Most of the interest from recent years is directed towards the micro RNA carried by exosomes. However, exosome-associated DNA in relation to pregnancy complications has not been truly studied yet. DNA, as an important cargo of exosomes, has been so far studied mostly in cancer research. This review collects all the known information on the topic of not only exosome-associated DNA but also some information on vesicles-associated DNA and the studies regarding the role of exosomes in pregnancy complications from recent years. It also suggests possible analysis of exosome-associated DNA in pregnancy from plasma and emphasizes the importance of such analysis for future investigations of pregnancy complications. A major obstacle to the advancement in this field is the proper uniformed technique for exosomes isolation. Similarly, the sensitivity of methods analyzing a small fraction of DNA, potentially fetal DNA, carried by exosomes is variable.

## 1. Introduction

Pregnancy complications are often associated with spontaneous preterm birth and might result in mortality or morbidity of the mother and/or the child. Despite significant improvements in monitoring and prevention in other areas of health care, the etiology of complications associated with pregnancy, such as preeclampsia (PE), intra-uterine growth retardation (IUGR), spontaneous abortion, or gestational diabetes mellitus (GDM), still remains unknown. However, a discovery of cell-free fetal DNA in the circulation of the mother represents the most important finding in this research field [1]. Since then, the noninvasive prenatal testing for chromosomal abnormalities can be performed using only a blood sample collected from the mother, which brings about new possibilities for prenatal diagnostics. Such a new technique is beneficial also when the increased concentrations of cell-free fetal DNA are detected in the circulation of mothers with various pregnancy complications [2]. Before this discovery, prenatal tests were performed by invasive methods such as amniocentesis or chorionic villus sampling. Unfortunately, these invasive procedures are associated with 1–2% fetal loss [3]. The noninvasive prenatal DNA diagnosis based on the analysis of cfDNA isolated from maternal plasma is rapidly evolving the research area; and using the latest methods for DNA analysis, various noninvasive prenatal tests are implemented into clinical practice. Currently, the determination of fetal gender, fetal Rhesus D (RhD) genotyping, aneuploidies, micro-deletions, and the detection of paternally-inherited monogenic disorders are available for pregnant women worldwide [4]. However, the clinical utility of cfDNA for noninvasive prenatal testing is even higher. In recent years, advanced investigation approaches have allowed to reconstruct the whole fetal genome [5,6] and methylome [7]. Based on the biological characteristic of cfDNA (its origin, fragment size and methylation), it presents also a potential new approach for pregnancy complication prediction and diagnosis [8].

Cell-free DNA (cfDNA) can be found in the circulation of every healthy individual. It has either nuclear or mitochondrial origin. It is released after cell death, such as necrosis or apoptosis [9], and is released as free fragments. Additionally, during inflammatory states, neutrophils produce net-like structures, which also cause the death of a neutrophil. These so-called neutrophil extracellular traps (NETs) are formed in order to kill pathogens. CfDNA, including histone proteins attached to the DNA, is incorporated into these structures [10]. CfDNA can also be released from the living cells through the process of exocytosis. Exocytosis is the process of releasing small vesicles, termed exosomes [11]. Exosomes are a subtype of extracellular vesicles (EVs). Recently, it was found that among other structures, EVs also contain DNA [12]. Since the DNA inside of vesicles is protected by the vesicular double membrane, EVs have been hypothesized to function in horizontal gene transfer.

Fetal cfDNA is produced mainly by the apoptosis of placental cells of the trophoblast [13]. It is produced during normal pregnancies; however, the process of apoptosis is likely increased during complicated pregnancies, due to increased oxidative stress and inflammatory response [14]. Phillipe et al. [15] were the first to hypothesize that fetal cfDNA could have a pro-inflammatory effect. Similar to bacterial DNA, fetal cfDNA is hypomethylated [2,16]. Toll-like receptor 9, expressed mainly on immune cells, is sensitive to hypomethylated DNA. The interaction of toll-like receptor 9 with fetal cfDNA triggers the innate immune response. This was experimentally proven on a mice model, in which free fetal DNA was injected into pregnant mice [16].

Living cells of the trophoblast also release EVs. It was published by Gupta et al. [17] that such vesicles contain both DNA and RNA. The aim of this review is to examine the role of fetal cfDNA from EVs in the pathogenesis of pregnancy complications. This review will present a summary of potential effects of fetal cfDNA in pregnancy complications; evidence of cfDNA being contained in EVs, specifically in exosomes; and a role of these exosomes in pregnancy complications. Since most recent cell-free nucleic acids papers focus on RNA, these studies are also mentioned; however, RNA is not a main focus of this review. The end this review concludes the possible techniques for the isolation of placental EVs and potential options for analysis of EVs that are beneficial for the field of pregnancy complications.

## 2. CfDNA and Pregnancy Complications

Preeclampsia (PE) is the most common of pregnancy complications. It is associated with the highest rate of morbidity and mortality among pregnant women worldwide. Clinically, it is characterized by hypertension, proteinuria and edema [18]. Spiral artery remodeling, altered angiogenesis and endothelial damage are believed to be involved in pathogenesis [19]. Recent meta-analysis [20] pointed out that cell-free fetal DNA is a predictive marker of both early and late onset PE. However, the most reliable detection time is at the beginning of the second trimester since the fetal fraction is the highest. It means that already in the first trimester, not much fetal DNA circulates in maternal blood compared to maternal DNA; and, on the other hand, in the third trimester, maternal DNA concentration increases with weight gain and concentration of fetal DNA becomes extinct [20]. Fetal RNA has also been studied as a potential biomarker of PE, especially at the beginning of the second trimester. A panel of specific mRNAs can serve as a predictor of PE by monitoring endothelial growth factor and endoglin, which are highly sensitive biomarkers [21,22]. Recently, the deportation of trophoblast was studied, and it was found that fetal trophoblast cells as well as immune cells were present in the lungs of the mother, which may have an immunomodulatory effect [23]. Interestingly, placental NETs have also been hypothesized to trigger the autoimmune reaction in PE [24]. Unfortunately, it is still not clear what causes PE and whether a high concentration of fetal cfDNA is a consequence or a cause.

Intrauterine growth restriction (IUGR) is characterized when the fetus does not achieve the normal growth due to placental causes, most often by placental insufficiency, which is associated either with hypoxia or with blood vessel morphology [25]. Other common causes of IUGR include maternal malnutrition as in vegan or vegetarian mothers [26] or exposure to toxic substances such as drugs or alcohol during pregnancy [27]. The causes cannot be exhaustively completed in this review, since except for the above-mentioned, more than 150 genetic disorders might be linked to IUGR [28]. Endocrine dysregulations along with other epigenetic factors might also contribute to IUGR. Nevertheless, the IUGR condition is often linked with PE. Maternal causes such as too young or too old maternal age; various maternal diseases and fetal causes such as chromosomal abnormalities; or multiple gestations and genetic factors are known key factors in IUGR. IUGR is another pregnancy complication as well as PE that might be associated with increased number of fetal cells and subsequently, an increased concentration of fetal nucleic acids in the circulation of a mother. Hahn et al. [29] described how fetal cfDNA might be used to study alterations in placentation, which is believed to be a cause of IUGR. However, the primary cause of altered placentation is probably due to a modification of maternal spiral arteries. Again, it is not clear whether high concentrations of cell-free fetal DNA contribute to the complication or if they result from it. A recent study discussed that even though cfDNA is higher during IUGR, it is important to consider the activity of deoxyribonuclease I, which is important for cfDNA elimination. This study showed that the activity of this enzyme is increased and therefore it is difficult to monitor cfDNA concentrations properly [30]. Considering the possible severe consequences of IUGR such as vascular, pulmonary, cardiac or neurological problems it is necessary to search for a predictor of IUGR and improvement of antenatal treatments [31].

Pregnancies are often complicated by spontaneous abortions, which are associated with increased stress or inflammation [32,33]. Although the terminology is not clear and standardized, spontaneous abortion may be defined as noninduced loss of an embryo or fetus or passage of conception products before the 20 weeks of gestation [34]. The major cause is chromosomal abnormalities [35]. Recently, it was shown that low progesterone metabolite concentrations contribute to spontaneous abortions due to triggering of inflammation [33,36]. Lim et al. [37] analyzed blood from 268 women and observed the association of spontaneous abortions with high fetal cfDNA concentrations. Similarly, Jakobsen et al. [38] observed that high concentrations of fetal cfDNA at the 25th week of pregnancy could predict the preterm delivery. The latest study of NETosis associated with spontaneous abortion showed that chorioamniotic NETs were found to be increased in women with chorioamnionitis and preterm delivery [39]. Similarly, it was recently found that cfDNA of both mitochondrial and nuclear origins were elevated in amniotic fluids in pregnancies complicated by preterm prelabor rupture of membranes [40]. Certain inflammatory cytokines and chemokines contribute to the pathogenesis of spontaneous abortions, which is associated with inflammatory mechanisms, and are possibly associated with cfDNA [36].

Gestational diabetes mellitus (GDM) is a spontaneous glucose intolerance developed during gestation. Chronic insulin resistance leads to β cells dysfunction, leading to glucotoxicity and later to macrosomia and lasting type II diabetes after delivery [41,42]. Nine miRNAs involved in placental and fetal development were discovered to be dysregulated during GDM. These miRNAs were of placental tissue origin [43]. However, other serum miRNAs were also found to be involved in the development of GDM and even possibly used as predictors of insulin resistance [44] or predictors of GDM in pregnancies associated with obesity [45,46]. CfDNA in the form of NETs has been associated with GDM [47]. A study by Stoikou et al. [48] showed that tumor necrosis factor alpha as a proinflammatory factor elevates NET formation in women with GDM compared to healthy controls. Also, Thurik et al. [49] pointed out the association between cfDNA and GDM in the first trimester of pregnancy. To the best of our knowledge, there are no other studies investigating cfDNA in GDM; however, such an association is often mentioned as a co-factor of other pregnancy complications.

Even though prenatal testing has been practiced for years already, using cfDNA as a marker from maternal blood is still in its initial studies. All the above-mentioned studies are only from recent years. However, this raises many questions and hypotheses that are yet to be answered or tested.

## 3. CfDNA Associated with Exosomes

EVs can be classified into three broad classes based on their size, endocytic origin, biogenesis and sedimentation properties—exosomes, microvesicles and apoptotic bodies. The exosome subclass is commonly defined as bilipid membrane-bound nanovesicles (size 40–120 nm in diameter) that are derived from multivesicular bodies. Under physiological and pathophysiological conditions, they are actively released from almost all types of cells into the extracellular space and body fluids [50,51]. Current research on circulating nucleic acids has shown that the circulating mRNA and miRNA molecules detected in plasma, serum and other biofluids are packaged in EVs [52,53], reflecting cells of origin and the disease state of the tissue. For example, exosomes from the plasma of pregnant preeclamptic women were analyzed, and it was demonstrated that their proteins [54] and microRNAs [55,56] could be used as biomarkers to predict PE. It was also found that preeclamptic placentas produce more extracellular vesicles in comparison to normal pregnancy [57,58], which could also be used as an indicator of the disease. For further comprehensive detailed information, there are several review articles available that deal with the biogenesis of all kinds of EVs [59,60,61,62].

Several studies have shown that exosomes also contain DNA molecules [63,64,65,66]. Thakur et al. revealed for the first time that tumor-derived exosomes contain double-stranded DNA, indicating that exosomal DNA reflects the mutational status of paternal tumor cells [66]. In other studies, double-stranded DNA was identified in exosomes isolated from the serum or plasma of pancreatic and prostate cancer patients [64,65]. This finding illustrates the translational potential of DNA isolated from exosomes as a circulating biomarker for the early detection of cancers and the monitoring of treatment response. Another newer study compared the concentrations of total DNA isolated from plasma and plasma exosomes and provided evidence that a large proportion (more than 90%) of plasma cfDNA is localized in exosomes rather than being truly free and circulating in plasma [67]. Apart from nuclear DNA, studies have also shown that cells release exosomes containing mitochondrial DNA (mtDNA) [68,69]. Guescini et al. reported that exosomes, constitutively released by glioblastoma cells and astrocytes, carry mtDNA, which can be further transferred between the cells [69]. The full mitochondrial genome was identified in plasma exosomes from patients with hormonal therapy-resistant metastatic breast cancer. It was further demonstrated that the horizontal transfer of mtDNA from vesicles acts as an oncogenic signal promoting an exit from the dormancy of therapy-induced cancer stem-like cells [70].

Despite the evidence of cfDNA association with exosomes, the discussion about its localization is still ongoing. Until now, most studies have been focused on intra-exosomal DNA. The exosomes have been shown to carry intra-vesicular DNA protected by a phospholipid bilayer membrane and the mutation status of this DNA was comparable to that of the cell origin [64,65,66,71]. In contrast, Shelke et al. [72] showed that DNase I-sensitive DNA could be associated with the outside of exosomes isolated from a human mast cell line. They have suggested that DNA can cause aggregation of these vesicles, possibly influencing their effects in recipient cells. Also, Nemeth et al. showed that ciprofloxacin-induced release of mitochondrial and chromosomal DNA is associated with the surface of exosomes [73]. Nevertheless, the above-mentioned studies showed that exosomes-associated DNA might be important in terms of physiological or pathophysiological states. CfDNA associated with EVs, especially exosomes, could be utilized as a biomarker in cancer, signal molecule or a molecule triggering the process of aggregation. However, the latest publication questions the real presence of DNA in exosomes and co-isolation of histone-bound DNA together with the isolation of exosomes [74]. It seems that the smallest vesicles, i.e., exosomes, do not carry DNA. On the contrary, DNA is released through autophagy, and endosomal mechanisms are independent from exosomal release [75].

## 4. Exosomes in Pregnancy Complications

Secretion of EVs, including exosomes, has also been reported in placental cells as a response to changes in the extracellular environment. The release of exosomes during pregnancy is modulated by particular features of the cellular microenvironment such as low oxygen tension (i.e., hypoxia) or high glucose concentration [76]. It was shown that placenta-derived exosomes regulate the migration and invasion of target cells; play an important role in intracellular communication; and potentially contribute to the placentation and development of maternal-fetal vascular exchange [77,78]. Placental exosomes are distinguished according to the placental alkaline phosphatase (PLAP) marker and are supposed to be released under placental dysfunctions [79]. Even animal models demonstrate fetal-maternal trafficking and signaling via exosomes [80]. It was shown that exosomes deliver paracrine cargo that signals labor and delivery of fetuses in mice [81]. Additionally, another study showed by quantitative proteomics that human maternal plasma exosomes physiology could possibly reflect the homeostasis in pregnancy and indicate preterm birth [82].

Placental-specific exosomes have been identified in maternal blood of healthy pregnant women. Beside PLAP, placental exosomes can be distinguished from maternally-derived exosomes by the presence of specific miRNAs [83,84,85]. Their concentration significantly increases during the first trimester of pregnancy and as early as the sixth gestational week [86], but the contribution of placental exosomes to total plasma exosomes and exosomes’ bioactivity decreases in late pregnancy [87]. Recent studies suggest a role of exosomes in pregnancy complications development. It has been shown that in pregnancy complications, such as PE and GDM, the number of secreted vesicles change [88,89,90]. A higher number of exosomes in the blood of pregnant women with early and late onset-PE compared with normal pregnancies was detected, which also suggests a possible pathophysiological role of placenta-derived exosomes in PE. The study showed a higher relative number of placental-derived exosomes in early onset-PE, but a lower relative number of placental-derived exosomes in late onset-PE in relation to total exosomes in maternal circulation [89]. Similarly, a 10-fold higher concentration of exosomes is present in the circulation of obese patients leading to proinflammatory pathways and insulin resistance when compared with normal weight patients [91].

As well as the placental origin, the origin of vesicles can be determined in more detail. The vesicles could be divided into embryo, oviduct, endometrial epithelium, and stroma-derived vesicles, all of which are involved in the communication with cells of trophoblast [92]. Extravillous trophoblast releases extracellular vesicles containing specifically human leukocyte antigen-G (HLA-G) that is only expressed in these cells. It was demonstrated that these vesicles are both exosomes and microvesicles [50,93]. HLA-G is known to protect the fetus from invasion by maternal immune system [94]; therefore, it is supposed that its function in exosomes is to signal and interact with responsible receptors [93]. It is believed that at the beginning of pregnancy EVs are mainly produced by cells of the endometrium, embryo and trophoblasts but later they are produced by syncytiotrophoblast cells, all having an immune-suppressing effect on the maternal immune system [95].

Many studies have focused on the differential expression of miRNAs in extracellular vesicles in placental tissue and blood from normal and preeclamptic pregnancies. They are summarized in a recently published review article of Chairello et al. [96]. A recent study showed various miRNAs cargo in maternal plasma exosomes obtained in the first trimester of pregnancy, which regulates different signaling pathways in pregnancy [97]. Also, Salomon et al. in their newest study of miRNA profiles of preeclamptic pregnant women suggested that the quantification of placenta-derived exosomes present in maternal blood and the measurement of specific miRNAs expression may improve our ability to identify asymptomatic women at risk for development of PE [98]. In addition to miRNAs, other potential molecular markers, such as placental proteins, angiogenic factors and fetal cfDNA, were investigated as potential predictive markers for PE development. The overview of all studies investigating small vesicles, exosomes, during pregnancy complications is summarized in Table 1.

To the best of our knowledge, there are no studies showing the presence of fetal cfDNA associated with exosomes circulating in maternal blood. Only a recent review described in detail the crosstalk between mother and the fetus, however, they discussed extracellular vesicles in general, not exosomes specifically [92]. A pilot study from our group showed that exosomes isolated from the blood of pregnant women contain not only DNA of maternal but also of fetal origin [123]. These results are raising new questions and provide the basis for further investigation of the localization of the vesicles and their function in healthy and complicated pregnancies. Nevertheless, the overview of studies regarding DNA in any relation to EVs or exosomes and pregnancy are summarized in Table 2.

## 5. Methods to Analyze cfDNA from Exosomes and Implications for Pregnancy Complications

High variability in the isolation as well as exosome characterization techniques used contributes to the diversity of published data [130]. Thakur et al. found that nucleic acid content in exosomes reflects the whole genomic DNA of parental cell lines [66]. They also compared different cancer cell lines suggesting that exosomal DNA could be used as a potential indicator of the disease reflecting its origin depending on the type of the cancer. It was already known that there is fetal DNA inside of exosomes isolated from placenta [119]. We hypothesize that exosome-associated fetal DNA in the circulation could also be used as a potential diagnostic marker for pregnancy complications. However, since no well-established method exists there have been problems with reproducibly isolating plasma exosomes. Also, the high abundance of plasma albumin interferes with several analyses of exosomes [131]. The most common isolation method is ultracentrifugation; however, size-exclusion chromatography [132] or different commercial kits [133,134,135] are also used.

Until now, the cancer research field has used cfDNA as a potential liquid biopsy biomarker to reflect the status of the organism [136]. There already exist protocols to obtain the highest yield of cfDNA since the concentration has to be high enough to make the analyses [137,138]. It was already published that 90% of cfDNA in plasma is hidden in exosomes [67]. So far, the common techniques of plasma exosome isolation followed by DNA analysis have been ExoQuick exosome precipitation solution [139], ExoLution Plus extraction technology [140] and differential centrifugation [141]. However, there have not been many studies analyzing fetal cfDNA in exosomes isolated from plasma. Other groups also hypothesize that fetal DNA could be carried in exosomes, which could therefore serve as suitable material for Y chromosome detection and fetal DNA quantification [127,142]. Our preliminary data showed that fetal DNA is packaged inside of exosomes and using the ultracentrifugation protocol for exosomes isolation we were able to quantify it [123]. Our study was the first one that attempted experimentally to quantify fetal DNA in exosomes. The results showed that the concentration of fetal DNA in plasma was 10 times higher than in plasma exosomes after the usage of DNase, that degraded any surface-bound DNA. The dynamics of fetal cfDNA in pregnancy is already known. Fetal DNA continuously rises throughout trimesters [143]. It is debatable whether cfDNA from the fetus is localized in exosomes or other vesicles. Perhaps more fetal DNA could be present in exosomes in the second trimester in comparison to other trimesters since it was found that the concentration of exosomes is the highest during this part of gestation [144]. However, potentially more fetal DNA could be localized inside of exosomes rather than being free, although, better optimization of isolation techniques is needed as well as more sensitive quantification methods. Extracellular RNA Communication Consortium has already started projects dealing with the isolation methods focused on RNA and of EVs in general [145]. Pregnancy complications are believed to be associated with an increased number of exosomes in maternal blood; therefore, it would be interesting to analyze plasma samples from women with PE, GDM or IUGR by a standardized method and obtain comprehensive information.

## 6. Conclusions

Every cell releases cfDNA. Fetal DNA is released from the cells of the placenta and circulates in the blood of the mother. Currently, it is known that fetal cfDNA could be used as a potential biomarker of several pregnancy complications. PE, IUGR, spontaneous abortions, or GDM are usually associated with increased concentrations of cell-free fetal DNA. Researchers in the field of exosomes have recently discovered that these small EVs also carry DNA. Such DNA is probably protected by their double membrane against the degradation by nucleases. As it is probably more stable, it could be used as a biomarker, and, therefore, is a source of information. However, the proper isolation method of placental exosomes followed by a proper isolation of vesicular fetal DNA needs to be optimized. As of now, the fetal cfDNA of exosomal origin is believed to have the potential to become a biomarker of pregnancy complications, along with the potential to explain the pathogenesis of various pregnancy complications in the future.

## Figures and Tables

**Table 1:** Chronological overview of studies regarding exosomes and pregnancy complications from years 2016–2018.

Study Year (Reference)	Pregnancy Complication	Material	Aim
Chang 2018 [99]	PE	Plasma	Antiangiogenic factors
Jayabalan 2018 [100,101]	GDM	Plasma, adipose tissue	Proteomics
Wang 2018 [102]	PE	Serum	miR-548c-5p
Menon 2018 [103]	Preterm birth	Plasma	Micro RNA
Luo 2018 [104]	IUGR	Umbilical cord blood	Micro RNA
Motawi 2018 [105]	PE	Umbilical cord blood and cell media	Micro RNA
Nair 2018 [106]	GDM	Placenta, plasma, skeletal muscle	Micro RNA
Hu 2018 [107]	PE	Urine	Expression of renal sodium transporters
Zhao 2018 [108]	IUGR, abortion	Plasma	Micro RNA
Miranda 2018 [109]	IUGR	Plasma	Basic characterization
Beretti 2018 [110]	Immune response	Amniotic fluid stem cell exosomes	Basic characterization
Shen 2018 [111]	PE	Serum	miR-155
Saez 2018 [112]	GDM	Plasma	Cargo
Biro 2017 [113]	Hypertension, PE	Plasma	Micro RNA
Ermini 2017 [114]	PE	Plasma	Cargo
Rodosthenous 2017 [115]	IUGR	Plasma	Micro RNA
Motta-Mejia 2017 [116]	PE	Plasma	Endothelial factors
Salomon 2017 [98]	PE	Plasma	Micro RNA
Truong 2017 [97]	PE, preterm birth	Plasma	Micro RNA
Elfeky 2017 [117]	Obesity	Plasma	Basic characterization
Shi 2017 [118]	GDM	Plasma	Micro RNA
Pillay 2016 [89]	PE	Plasma	Concentration
Sheller 2016 [119]	Preterm birth	Amniotic membrane	Cargo
Gysler 2016 [120]	Autoimmune disorders	Plasma	Micro RNA
Ospina-Prieto 2016 [121]	PE	Trophoblast cells	miRNA-144
Sandrim 2016 [56]	PE	Plasma	miR-885-5p
Salomon 2016 [90]	GDM	Plasma	Concentration
Panfoli 2016 [122]	Preterm birth	Umbilical cord cells	Characterization

PE—preeclampsia, IUGR—intra-uterine growth retardation, GDM—gestational diabetes mellitus.

**Table 2:** Chronological overview of studies regarding DNA and pregnancy complications.

Study Year (Reference)	Pregnancy Complication	Material	Aim
Fernando 2018 [124]	Pregnant vs. Non-pregnant	Plasma	Fragment size pattern of cfDNA
Sheller-Miller 2017 [125]	Term labor	Amnion ephithelial cells	Cargo
Tong 2017 [126]	PE	Placental explants	Concentration of EVs
Sheller 2016 [119]	Term delivery	Amnion ephithelial cells	Cargo
Orozco 2009 [127]	PE	Plasma	EVs containing DNA concentrations
Orozco 2008 [128]	PE	Extravillous trophoblast and plasma	EVs containing DNA basic characterization
Gupta 2005 [129]	PE	Placental explants and neutrophils	NETs formation

## Abbreviations

CfDNA	cell-free DNA
NETs	neutrophil extracellular traps
EVs	extracellular vesicles
